# Renewable Human Cell Model for Type 1 Diabetes Research: EndoC-*β*H5/HUVEC Coculture Spheroids

**DOI:** 10.1155/2023/6610007

**Published:** 2023-12-21

**Authors:** James M. Porter, Michael Yitayew, Maryam Tabrizian

**Affiliations:** ^1^Department of Biological and Biomedical Engineering, Faculty of Medicine and Health Sciences, McGill University, Montreal, QC, Canada H3A 0G4; ^2^Faculty of Dental Medicine and Oral Health Sciences, McGill University, Montreal, QC, Canada H3A 1G1

## Abstract

*In vitro* drug screening for type 1 diabetes therapies has largely been conducted on human organ donor islets for proof of efficacy. While native islets are the ultimate target of these drugs (either *in situ* or for transplantation), significant benefit can be difficult to ascertain due to the highly heterogeneous nature of individual donors and the overall scarcity of human islets for research. We present an *in vitro* coculture model based on immortalized insulin-producing beta-cell lines with human endothelial cells in 3D spheroids that aims to recapitulate the islet morphology in an effort towards developing a standardized cell model for *in vitro* diabetes research. Human insulin-producing immortalized EndoC-*β*H5 cells are cocultured with human endothelial cells in varying ratios to evaluate 3D cell culture models for type 1 diabetes research. Insulin secretion, metabolic activity, live cell fluorescence staining, and gene expression assays were used to compare the viability and functionality of spheroids composed of 100% beta-cells, 1 : 1 beta-cell/endothelial, and 1 : 3 beta-cell/endothelial. Monoculture and *β*H5/HUVEC cocultures formed compact spheroids within 7 days, with average diameter ~140 *μ*m. This pilot study indicated that stimulated insulin release from 0 to 20 mM glucose increased from ~8-fold for monoculture and 1 : 1 coculture spheroids to over 20-fold for 1 : 3 EndoC-*β*H5/HUVEC spheroids. Metabolic activity was also ~12% higher in the 1 : 3 EndoC-*β*H5/HUVEC group compared to other groups. Stimulating monoculture beta-cell spheroids with 20 mM glucose +1 *μ*g/mL glycine-modified INGAP-P increased the insulin stimulation index ~2-fold compared to glucose alone. Considering their availability and consistent phenotype, EndoC-*β*H5-based spheroids present a useful 3D cell model for in vitro testing and drug screening applications.

## 1. Introduction

Human islet transplantation is a minimally invasive procedure which offers the hope for a one-time curative treatment for type 1 diabetes. Harvesting functional insulin-producing cells from recently deceased donors provides a replacement strategy for beta-cells lost to autoimmune destruction. While a promising treatment option, long-term success of islet transplants could be improved, with recipients who maintain stable blood glycemia dropping from 87.5% to 71% after one year, with significant decline thereafter [1, 2]. The most direct threats to successful islet transplantation are the instant blood-mediated inflammatory response (IBMIR) and hypoxia due to difficulty establishing sufficient vascular connectivity [3]. The importance of mitigating immune rejection by donor/recipient blood type matching further complicates the existing shortage of donor cells. Lifelong immunosuppressive medication is administered after transplant, which increases the risk of infection and burdens the medical system.

Accessory cells, such as endothelial, mesenchymal, and other nonbeta islet cells, can benefit insulin secretion and vascular connectivity [4]. Culturing cells as 3D “spheroids” provides an avenue for *in vitro* functionality testing and drug screening. Due to limited donors, priority for successful isolations is given to transplantation, creating a severe shortage of primary islets for research. Islet-like cell clusters recapitulate native morphology and insulin production, providing freedom from timing of availability and number of biological replicates. Forming spheroids with fewer cell types reduces background noise in cellular crosstalk investigations. Incorporating accessory cells in models supports beta-cell grafts, by secreting performance-enhancing proteins [5, 6]. Recently, human umbilical vein endothelial cells (HUVECs) have been shown to improve vascularization and nutrient distribution when cotransplanted with islets [7–9]. Endothelial cells improve beta-cell survival by expressing basement membrane proteins and send paracrine signals through the bloodstream, playing a critical role in supporting insulin gene expression and glucose-stimulated insulin secretion (GSIS) [10, 11]. Protein-secreting accessory cells may therefore be superior to adding molecular factors themselves [12]. In the future, autologous endothelial cells could help reduce the foreign body immune response [13]. Endothelial cells regulate immune mediators, acting to protect the glucose sensing mechanism and control permeability on the outer boundaries of islets [14]. Factors such as INGAP-P, exendin-4, or IBMX promote insulin secretion, maximizing the effect of smaller beta-cell populations [15–17]. Identifying therapeutic compounds relies on stimulating human islets, posing challenges due to their short *in vitro* lifetimes, lack of proliferation, and significant donor-to-donor variation [18].

The scarcity of human islets for research coupled with functional heterogeneity between isolations makes drug screening costly and inefficient. Here, we present a 3D cell model to facilitate *in vitro* screening for type 1 diabetes (T1D) research. We first validate the spheroid formation protocol using the proliferative mouse insulinoma MIN6 line and then focus on coculturing HUVECs with the nonproliferative EndoC-*β*H5 human beta-cell line. The MIN6 line has been shown to increase the insulin response to glucose 2-fold in 3D spheroids compared to monolayer culture, providing a renewable *in vitro* model for animal islet testing [19]. EndoC-*β*H5 is the latest development of Human Cell Design, who first pioneered the immortalized human EndoC-*β*H1 cell line. While the insulin release of EndoC-*β*H1 cells can be suppressed using mannoheptulose, continued proliferation would present issues in terms of graft stability and hypoglycemia [20, 21]. To stop replication while promoting maturation, the EndoC-*β*H5 line has had immortality reversed, which can be accomplished by excision of hTERT and SV40LT, or even temporary inactivation of SV40LT mRNA [22]. EndoC-*β*H1 cells have shown improved function when combined with supporting cells in spheroids [17, 20, 23]. The nonreplicating, newer generation EndoC-*β*H5 line, showing a higher response to glucose and insulin secretagogues, has also recently been validated for drug screening applications [24]. Cocultured spheroids with interspersed endothelial cells have increased cell-cell communication compared to layered shell structures [25]. In this study, we explore the impact of varying ratios of low-passage HUVECs to nonproliferative EndoC-*β*H5 cells in 3D spheroids, aiming to establish a robust model for T1D research. This comparative study evaluates “pseudoislet” spheroid groups by GSIS, gene expression, metabolic activity, and immunofluorescence.

## 2. Methods and Materials

### 2.1. Cell Culture

Human umbilical vein endothelial cells (HUVECs) purchased from Lonza (Cedarlane Labs, ON, Canada) were thawed and seeded in T75 tissue culture flasks at a density of 300,000 cells per flask. Supplier prescreening-verified HUVECs showed trypan blue viability of 85-90% and 98% CD31/CD105 double positive. HUVECs were cultured in EGM-2 endothelial cell growth media and supplemental BulletKit (Cedarlane Labs, ON, Canada). MIN6 mouse beta-cells (kindly gifted by Prof. Hoesli from McGill University) were seeded at 2-3 million cells per flask. The human beta-cell line EndoC-*β*H5 (Human Cell Design, France) was seeded at 80,000 cells/cm^2^ and maintained without proliferation, changing media weekly. Low-passage HUVECs (p4-p8) and immortal MIN6 cells (p30-p40) were used for spheroid formation [26]. Cells were trypsinized using 0.05% trypsin with EDTA and resuspended at 2 million cells/mL for seeding onto agarose molds. To prepare the molds, 0.9% (*w*/*v*) saline solution was mixed by dissolving NaCl powder in D.I. H_2_O to 9 mg/mL and then filtered by 0.2 *μ*m syringe filter. 500 mg of agarose powder was then dissolved into 20 mL of the filtered saline solution in a 50 mL falcon tube, resulting in a 2.5% agarose (*w*/*v*) mixture. The mixture was heated in a microwave at 10 s increments until fully dissolved. 300 *μ*L of warm agarose was pipetted into MicroTissue 3D Petri dish micromold casts (Sigma-Aldrich, ON, Canada). After cooling for 10 min, the agarose forms a gel and can be removed from the cast and stored at 8°C in a saline solution.

### 2.2. Spheroid Formation

As cells expanded in monolayer culture, agarose microwell molds were prepared for spheroid formation. Molds were precondition by immersion in growth media in a 24-well plate for 30 min. The media was then removed, and cells were pipetted in a 130 *μ*L suspension onto the premade molds. To attain 1000 cells in each of the 256 wells of the gel microarray, a density of 256 k cells/130 *μ*L (~2 million cells/mL) was used.

Based on previous experiments coculturing human islets with HUVECs and MIN6 insulinomas with HUVECs at beta/endothelial cell ratios of 1 : 2 and 1 : 6, respectively, we opted for 1 : 1 and 1 : 3 in our coculture groups [27, 28]. For 1 : 1 beta-cell/HUVEC coculture spheroids, 62.5 *μ*L of beta-cells and 62.5 *μ*L of HUVEC containing cell suspensions were added, respectively, at the appropriate density. For 1 : 3 beta-cell/HUVEC constructs, 32.5 *μ*L of beta-cell suspension is added to the mold, along with 97.5 *μ*L HUVEC cell suspension (table S1).

The initial seeding was left undisturbed for 4 hours to allow the cells to sediment into the microwells and begin aggregating, at which point 1.3 mL of media was added to each well of the 12-well plate to submerge the molds and incubated at 37°C. Growth medium was changed every 3 days, as per normal culture protocols. Coculture spheroids were immersed in a 50 : 50 blend of Ulti-*β* EndoC-*β*H5 cell and EGM-2 HUVEC media. Over 5-7-day cells aggregated into compact spheroids and could be flushed from the molds and resuspended (visual depiction given by Guo et al. [29]).

### 2.3. Immunofluorescent Confocal Imaging

Live/dead staining for fluorescence imaging was performed using the Biotium Viability/Cytotoxicity Assay Kit (for animal, VWR, CA, USA). The green (live) dye consisted of an elastase substrate, which was cleaved into fluorescent calcein and only remained if the cell membrane was intact. The red (dead) dye was Ethidium Homodimer-III (EthD-III), a membrane-impermeable DNA dye, only penetrating cells when the cell wall was compromised. Spheroids were prepared for imaging by washing twice with PBS. Staining media was 2.5 *μ*L calcein AM (hydrolyzed, pH 8) and 10 *μ*L EthD-III in PBS (5 mL total volume). Spheroids immersed in staining media were shielded from light and incubated at 37°C for 30 min before imaging. Images were taken using the LSM 710 confocal scanning microscope (excitation wavelengths: 488 nm for live and 543 nm for dead, Zeiss Axio Observer).

For cell-specific staining, spheroid samples were washed twice with PBS and fixed in 3.5% PFA for 10 min at 37°C, followed by two more PBS rinsing steps to remove the fixative and stored in the dark at 4°C until staining. Samples were washed twice in tris-buffered saline (TBS; 50 mM Tris, 150 mM NaCl) + 0.05% Tween-20 and blocked for 30 min using TBS + 3% BSA. Samples were washed twice in TBS + 0.05% Tween-20 and once in TBS before staining overnight with the CD31-Alexa Fluor 488 nm conjugate (Biotium, CA, USA) and insulin primary antibody, both diluted to 1 *μ*g/mL, targeting HUVECs and beta-cells, respectively. Samples were washed 3× in TBS + 0.05% Tween-20, and Alexa Fluor 647 nm secondary antibody was used to stain for 1 hour and washed a final 3× in TBS + 0.05% Tween-20. As a negative control, the 488 nm secondary antibody was incubated with samples without the insulin primary antibody (no fluorescence was observed). Image reconstruction and analysis were performed using the Zen Microscope Software by Zeiss.

For cell tracker staining, adherent monolayer cells were dyed prior to spheroid formation, in contrast to target-specific antibody staining of fully formed spheroids. T75 flasks containing either EndoC-*β*H5 or HUVEC cells were incubated with the CellTracker red or blue dye, respectively (Invitrogen, ThermoFisher Scientific, ON, Canada), for 30 min and then placed back in growth media until imaging.

### 2.4. Metabolic Activity Assay

Cell viability was measured using the AlamarBlue reagent from Invitrogen (ThermoFisher Scientific, ON, Canada), based on the reduction of resazurin to resorufin by healthy cells. The assay reagent was diluted 1 : 9 in media and incubated with living cells for 4 hours. Following incubation with living cells, the test reagent was collected and replaced with fresh growth media. The percent reduction of the test reagent was calculated by measuring the absorbance or optical density at both 570 nm and 600 nm:

(1) $$\begin{array}{c} \%\text{Reduction}=\frac{\left(\left(\epsilon_{\text{OX}}\left(600\text{ nm}\right)*A_{1}\left(570\text{ nm}\right)\right.-\left(\epsilon_{\text{OX}}\left(570\right)*A_{1}\left(600\text{ nm}\right)\right)\right.}{\left(\left(\epsilon_{\text{RED}}\left(570\text{ nm}\right)*A_{2}\left(600\text{ nm}\right)\right.-\left(\epsilon_{\text{RED}}\left(600\text{ nm}\right)*A_{2}\left(570\text{ nm}\right)\right)\right.}*100\%, \end{array}$$

where *A*_1_ denotes the sampled supernatant and *A*_2_ the negative control comprised of the serum-free media diluted AlamarBlue reagent at a ratio of 1 : 9. The molar extinction coefficients for the 2 wavelengths are *ϵ*_OX_(600 nm) = 117,216, *ϵ*_OX_(570 nm) = 80,586, *ϵ*_RED_(570 nm) = 155,677 , and *ϵ*_RED_(600 nm) = 14,652, respectively. Both samples and controls were measured for optical density at 570 nm and 600 nm incident light for determination of the percent reduction of AlamarBlue reagent.

### 2.5. Glucose-Stimulated Insulin Secretion (GSIS)

After allowing cells to compact for 7 days, spheroids are flushed from the molds by gentle pipetting. Prior to glucose stimulation, cells were placed in a low-glucose starvation media for 24 hours. The test stimulation was carried out in Krebs's buffered salt solution, prepared at 0 mM and 20 mM glucose for “low” and “high” solutions, respectively. For EndoC-*β*H5, high- and low-glucose solutions were made using *β*-Krebs purchased from Human Cell Design (Toulouse, France). Upon recovery from starvation media, cells are washed for 1 hour in the low-glucose Krebs and then incubated for one hour in low glucose, followed by 1 hour in high glucose, at 37°C. Supernatant from each spheroid group, having 0, 50, or 75% HUVECs, was sampled in triplicate for insulin measurement. Once the stimulation was complete, the spheroids were lysed and stored at -80°C for mRNA quantification and gene expression analysis. Insulin secretion was quantified using the Mercodia human insulin ELISA kit (Cedarlane Labs, ON, Canada).

### 2.6. Beta-Cell Gene Expression Assay (RT-qPCR)

Following GSIS testing, cells were lysed for gene analysis. Nuclear mRNA was extracted and purified using the Invitrogen PureLink RNA Mini Kit (ThermoFisher Scientific, ON, Canada) protocol and evaluated by NanoDrop quantification. Purified RNA samples were stored at −80°C until gene expression analysis using the CFX Opus Real-Time PCR system (BioRad, ON, Canada). Primers specific to beta-cell genes were chosen to evaluate the spheroids in terms of insulin production and beta-cell maturation. Forward and reverse sequences for reference and target genes were obtained from Integrated DNA Technologies (IA, USA) and prepared for testing using the Luna OneStep reaction kit (New England Biolabs, MA, USA). mRNA was reverse transcribed into cDNA, amplified by thermal cycling, and detected by SYBR green fluorescence. Target gene expression was quantified relative to the housekeeping gene glyceraldehyde-3-phosphate dehydrogenase (GAPDH) within each group and normalized to the monoculture spheroid control group for fold change analysis (*R* = 2^−ddCt^). Glut2 is a transmembrane glucose transporter, and PDX1 is involved with endoplasmic reticulum health and function, while MAFA is a beta-cell specific transcriptional activator (primer sequences and gene descriptions shown in table S2, visual diagram in figure S4) [30].

## 3. Results

### 3.1. Spheroid Formation and Viability Assay

**Figure 1:**
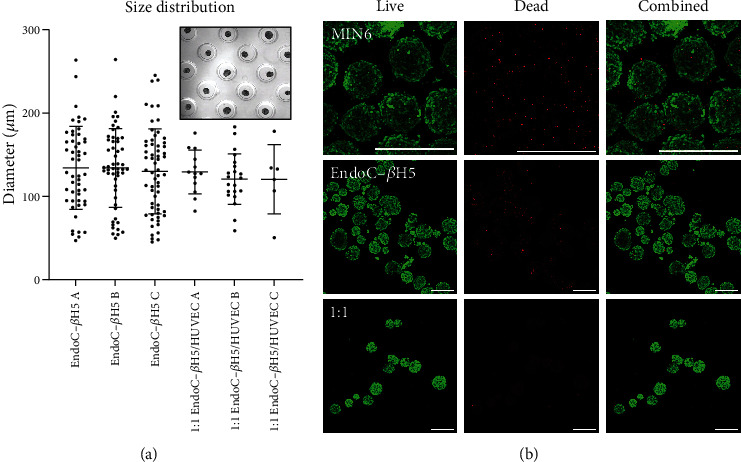
(a) Diameters of individual spheroids in each of 3 batches for monoculture and 1 : 1 EndoC-*β*H5/HUVEC cocultures (inset: cells in agarose microwells, center-to-center spacing is 580 *μ*m). (b) Live/dead confocal imaging for MIN6 monoculture, EndoC-*β*H5 monoculture, and 1 : 1 EndoC-*β*H5/HUVEC coculture spheroids. Scale bar is 250 *μ*m.

Figure 1(a) shows the ImageJ-quantified diameter of spheroids after 7 days of sedimentation. Monoculture aggregates compacted faster than cocultures during the first 5 days. However, monoculture and 1 : 1 coculture spheroids did not have significant differences in diameter or cross-sectional area by day 7, respectively. While 1 : 1 coculture spheroids exhibited a slightly narrower distribution, all groups fell under the target of 150 *μ*m for “average” human islets of 1 IEQ. Cross-sectional spheroid areas are shown in figure S1.

The live (green, 488 nm) and dead (red, 647 nm) staining and combined images for different spheroid groups acquired by confocal fluorescence imaging on day 12 after spheroid seeding are shown in Figure 1(b). Recombined *Z*-stack images show both monoculture EndoC-*β*H5 and 1 : 1 cocultures display a compact structure with spheroidal morphology and few dead cells. Monoculture MIN6 spheroids appeared less tightly bound.

ImageJ quantification of live/dead cell images was used to quantify viability (Figure S2A). Percent viability is calculated by the number of live cells divided by the total cell number. MIN6, EndoC-*β*H5, and 1 : 1 (*β*H5/HUVEC) spheroid groups showed statistically similar viabilities of ~90%. Figure S2B shows the metabolic activity of MIN6 monoculture and MIN6/HUVEC coculture spheroids at days 3, 5, and 8 after formation.

**Figure 2:**
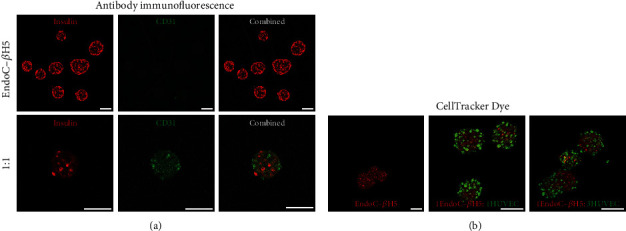
Spheroids stained with fluorescent antibodies specific to insulin in EndoC-*β*H5 (red) and CD31 in HUVECs (green). (b) CellTracker cell dye on EndoC-*β*H5 (red) and HUVECs (green), which are stained in monolayer culture prior to combined spheroid formation. Scale bar is 100 *μ*m.

Using cell-specific stains for insulin and CD31, the beta and endothelial cells could each be resolved. Confocal imaging using separate captures for the 2 dyes (647 nm for insulin and 488 nm for CD31) is shown for 100% EndoC-*β*H5 monoculture and 1 : 1 EndoC-*β*H5/HUVEC spheroids in Figure 2(a). CellTracker dye stain of EndoC-*β*H5 (red) and HUVEC (green) monolayer cells prior to spheroid formation shows the incorporation of both cell types within the construct (Figure 2(b)). Total fluorescent intensity quantification revealed HUVEC/beta-cell ratios of 0.9 and 1.4 for the 1 : 1 and 1 : 3 groups, respectively. Cell-specific staining including nuclear staining for 1 : 1 coculture spheroids is shown in figure S3.

### 3.2. Glucose-Stimulated Insulin Secretion

**Figure 3:**
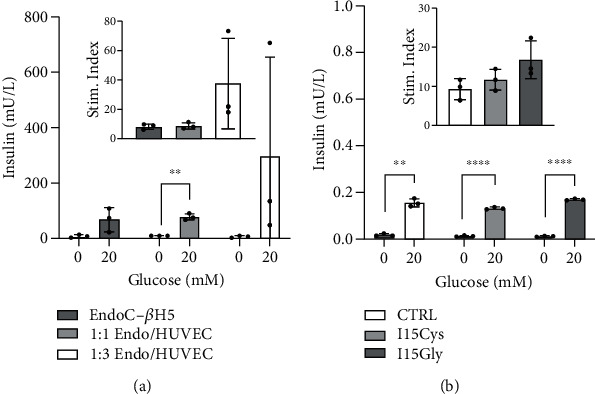
(a) ELISA quantification of insulin secretion in response to low (0 mM) and high (20 mM) glucose (inset: stimulation). (b) Insulin response to low (0 mM) and high (20 mM) glucose alone and with 1 *μ*g/mL INGAP-P variants I15Cys or I15Gly (inset: stimulation index).

After 7 days of culture in molds, spheroids were recovered and starved for 24 hours in low-glucose media before stimulation.  Figure 3 shows the ELISA-quantified insulin concentration of the Krebs glucose buffer following incubation with different spheroid groups.  Figure 3(a) presents the insulin concentrations measured from supernatant following incubation of the spheroids in low- and high-glucose stimulation buffer. The inset shows corresponding stimulation indices, calculated as the ratio of high to low insulin concentrations. Cocultured EndoC-*β*H5/HUVEC (1 : 1) spheroids displayed the highest consistency among triplicates and the most statistical significance in insulin production, with *p* = 0.0085 between high and low values (denoted as “^∗∗^”). The 1 : 3 group exhibited the highest insulin release and stimulation index, though were not significantly different due to variation among triplicates.  Figure 3(b) shows the response of 100% EndoC-*β*H5 spheroids to 0 mM and 20 mM glucose containing 1 *μ*g/mL INGAP-P modified with cysteine (I15Cys) or glycine (I15Gly) residues, with inset showing the corresponding stimulation indices. Details of synthetic INGAP-P peptide screening are covered in our previous work [15]. Peptides were custom synthesized as analogues of the 15 amino acid sequence, INGAP-P, to find stable conformations with increased stimulatory effect. All 3 groups showed a marked insulin response to elevated glucose (~10-15-fold). While exposing EndoC-*β*H5 spheroids to I15Gly nearly doubled the stimulation index, this alone is not enough to conclude the peptide's beneficial properties. The reproducible stimulation response to glucose, however, demonstrates the usefulness of this cell model for future studies. Investigating a range of dose concentration and exposure time would help identify peptides or other agents for stimulatory effects.

### 3.3. Metabolic Activity and Gene Expression

**Figure 4:**
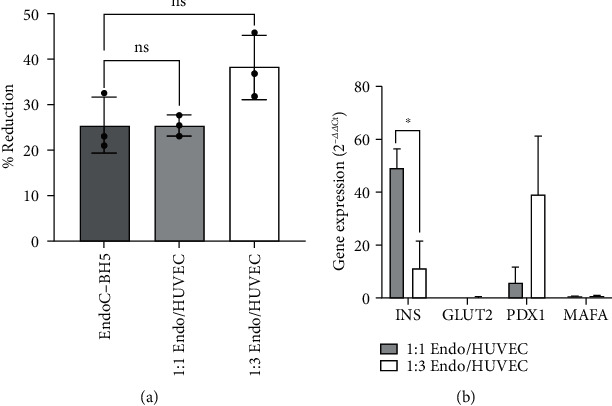
(a) Metabolic activity, shown as percent reduction of the AlamarBlue reagent. 1EndoC-*β*H5/3HUVEC spheroids showed increased activity (*p* = 0.077). (b) RT-qPCR quantification of beta-cell-specific mRNA targets, shown as *R* = 2^−ddCt^, relative to the GAPDH housekeeping gene, with monoculture spheroids used as the control group. A paired *t* test found significant difference between the insulin expression of 1 : 1 and 1 : 3 coculture groups (*p* = 0.02).

Figure 4(a) displays the metabolic activity of the 3 spheroid groups as measured by the AlamarBlue assay, and 4B the gene expression values of target genes quantified relative to the internal GAPDH reference. The monoculture and 1 : 1 coculture groups had a similar percentage reduction, whereas the 1 : 3 EndoC-*β*H5/HUVEC group had a higher metabolic activity, compared to other groups (*p* = 0.077). The Livak-Schmittgen value, *R* = 2^−ddCt^, gives the fold change for mRNA levels in 1 : 1 and 1 : 3 coculture groups, normalized against the monoculture control [31]. Both coculture spheroid groups displayed insulin and PDX1 upregulation compared to monoculture spheroids, with significant increases of insulin expression in the 1 : 1 group compared to 1 : 3.

## 4. Discussion

Maximizing the insulin production and survival of implanted cells are critical to islet graft efficacy and longevity. Our previous work highlighted the variability in drug screening studies on human islets due to individual differences between donors [15]. Age, BMI, comorbidities, and drug history all act to confound the accuracy of studies with limited donor access. Alternatively, immortalized cell lines provide a stable phenotype for greater consistency and an unlimited cell supply. 3D culture enables studying morphological effects on paracrine interactions. Endothelial cells cultured in support of insulin-producing beta-cells can provide molecular stimulation and regulation of immune mediators and induce vascularization [5, 32, 33]. Mutually beneficial signaling occurs as beta-cells release angiogenic VEGF-A, and islet ECs in turn produce endothelin-1 and endothelin-3, which enhance GSIS [14]. To investigate the effect of endothelial cells on beta-cells, we studied three groups of spheroids in varying proportions of the two cell types. The first group, a 100% EndoC-*β*H5 monoculture, served as negative control for the cocultured spheroid groups at 1 : 1 and 1 : 3 beta-cell/EC ratios. The total cell number was held constant at 1000 cells/well.

3D structures increase cell-cell contact and improve cell function. The mouse insulinoma MIN6 cell line has consistent proliferation into high passages (<80) and releases mouse insulin in response to glucose [26]. In a GSIS assay, beta-cells are immersed in a low-glucose (or basal) media, which is then replaced with a high-glucose stimulation media. Supernatant sampling after 1-hour incubation in each condition provides the fold change and total insulin output. MIN6 spheroids formed in microwells demonstrated improved insulin release compared to monolayer cultures [34]. Increased cell-cell communication may play a role in the concerted insulin response. Previous spheroid studies combining MIN6 cells with mouse aortic ECs or with NIH3T3 fibroblasts showed increased insulin secretion and gene expression, compared to MIN6 alone [28, 35]. Following promising indications in the literature, we used MIN6 cells to test our spheroid formation and HUVEC coculture process as their proliferative nature facilitates multiple rounds of optimization. While pure MIN6 groups formed spheroids with acceptable morphology, mixed groups containing HUVECs were more dispersed and disaggregated upon handling. As GSIS responses were variable across replicates, we focused on EndoC-*β*H5 for stimulation studies. This human beta-cell line has shown improved insulin response to glucose (>6-fold change) and is a species-match for coculture with human endothelial cells. Here, we constructed EndoC-*β*H5/HUVEC spheroids (“human pseudoislets”) for evaluation as an *in vitro* model for T1D screening studies. Spheroid formation has been shown to increase the insulin response to glucose of EndoC-*β*H3 cells, and the presence of islet endothelial cells improved insulin release in EndoC-*β*H1 spheroids [23, 36]. Heterocellular spheroids with varying proportions of EndoC-*β*H5/HUVEC were evaluated for the effect of directly incorporating endothelial cells into the model. Interspersed beta-cell/HUVEC morphology was chosen over core/shell designs to reflect the capillary networks throughout human islets.

The use of preformed agarose molds allowed for precise formation of 3D spheroids without the need for microfabrication or photolithography used for PDMS-based microfluidic channels. We formed spheroids of controlled size and shape by seeding 1000 cells into each microwell of the array for aggregation. Acceleration of spheroid formation can be achieved by centrifugation or actuation of magnetically functionalized beads, though suspension cells can still form compact and robust spheroids by gravity over 5-7 days [37]. MIN6 cells have shown a synergistic effect and increased insulin secretion when cultured with HUVECs, compared to monoculture MIN6 spheroids [28]. 250 MIN6 cells were cultured for 1 day, and then, 1500 HUVECs were added in suspension before centrifugation at 150 g for 30 s. Here, we added beta-cells and HUVECs in suspension at the same initial time point, allowing sedimentation to occur by gravity. Due to this softer aggregation, we started with lower beta-cell/HUVEC ratios of 1 : 1 and 1 : 3, as opposed to the 1 : 5 used with centrifugation. Higher endothelial content was seen to result in increased cellular debris and more loosely bound aggregates.

Immunofluorescence staining revealed consistent spherical morphology, good viability (~90%), and metabolic activity among EndoC-*β*H5 coculture and monoculture spheroid groups. Metabolic activity as determined by the AlamarBlue cell viability assay showed an increased % reduction for the 1 : 3 group, with the highest proportion of endothelial to beta-cells, as compared to monoculture spheroids (*p* = 0.077). No change was seen in the 1 : 1 spheroid group. A proliferation assay could help to distinguish metabolism between groups if there is continued expansion of HUVECs. Spheroids with higher endothelial cell content also showed increased insulin release per beta-cell, yet further statistics are required to determine significance. Stimulation in smaller volumes with lower spheroid numbers could produce a comparable response with less consumption of nonproliferating EndoC-*β*H5 cells.

Our previous work has shown the potential of INGAP-P-based peptides to improve insulin secretion and expression of certain beta-cell gene mRNAs but dealt with high variability among human donors [15]. Here, we selected I15Cys and I15Gly for their potential to improve insulin secretion, possibly in conjunction with upregulation of PDX1 expression. While the increased stimulation indices seen with EndoC-*β*H5 spheroids exposed to I15Cys and particularly I15Gly are not sufficient to conclude their use as insulin secretagogues, the EndoC-*β*H5 3D cell models demonstrate improved performance for in vitro drug discovery testing, compared to human donor islets. Further studies should determine the effects of varying peptide dosage concentration and exposure time.

Beta-cell-specific gene markers related to the insulin secretion pathway were analyzed for each spheroid group following glucose stimulation. Insulin, GLUT2, PDX1, and MAFA were quantified relative to the glyceraldehyde-3-phosphate dehydrogenase (GAPDH) housekeeping gene in each group. While GAPDH expression generally remains constant in response to different treatments, some variation may occur among different human donors [38, 39]. GAPDH has been found to have more stable expression compared to 18 s RNA, beta-actin, and other reference genes in EndoC-*β*H5 cells when quantified by RT-qPCR [40]. Here, by using these “deimmortalized” clone cells, we expect little to no variation across housekeeping genes in different groups. In our study, the 1 : 1 coculture models displayed a significant upregulation of the insulin gene compared to the 1 : 3 group, and both coculture groups show increased insulin and PDX1 compared to monoculture controls. In the context of a T1D research model, future studies should evaluate any changes in gene and protein expression in response to proinflammatory cytokines. Coculture spheroids of varying composition could be exposed to interleukin-1*β*, interferon-*γ*, and tumor necrosis factor-*α*, for instance, as has been done with adherent EndoC-*β*H5 cells in monoculture [40].

Researchers have shown endothelial signaling occurs via secretion of laminins and basement membrane proteins which enhance proliferation and upregulate insulin gene expression in beta-cells [41]. Islet endothelial cells produced the ECM protein *β*1 integrin when cocultured with native beta-cells, the blockade of which reduced insulin gene expression and increased insulin secretion at basal glucose levels [6]. PDX1 expression is known to be reduced in apoptotic beta-cells due to loss of insulin and insulin-like growth factor (IGF) signaling associated with endoplasmic reticulum stress [42, 43]. Reduced expression of PDX1 and MafA can occur in response to oxidative stress, as seen in the early pathogenesis of T1D [44]. Loss of insulin and MafA expression is also associated with loss of beta-cell identity through dedifferentiation [45]. Since GLUT2 functions as a glucose transporter, limited mRNA expression indicates lower activation of the pathway required for pancreatic beta-cells to initiate the insulin response [46]. As these genes are involved with differentiation and maturation, the upregulation of insulin and PDX1 indicates a strong expression of the beta-cell phenotype [21, 47]. Stimulation of the transmembrane glucose transport Glut2 would likely contribute to improved performance in triggering insulin release. Future studies could expand the gene panel to include expression markers of HUVEC integration and function, such as primers for CD31, CD105, VE-cadherin, as well as testing for VEGF-*α* and endothelin protein production [48].

A limitation to these experiments, and the field in general, is the lack of a renewable human alpha-cell line for incorporation within the spheroids. A promising step forward, these 3D cocultures cell are vastly simplified models of native human pancreatic islets. Current efforts employ fluorescence activated cell sorting (FACS) to reaggregate cells from previously dissociated islets; however, stable alpha lines for in vitro culture are not widely established or available at this time [49, 50]. Furthermore, although live/dead confocal imaging confirmed viability throughout the 3D constructs, it would be instructive to employ perfused culture chambers to investigate the dynamic perfusion of oxygen and nutrients through the spheroids over time and in varying levels of hypoxia. Fluid shear stress matching physiological levels would also allow for accurate determination of the real-time secretion response to stimuli.

## 5. Conclusion

The human insulin-secreting EndoC-*β*H5 line outperformed mouse-derived MIN6 in spheroid stimulation index. Cocultured spheroids containing beta-cells and HUVECs displayed increased insulin secretion and metabolic activity, compared to monoculture. Reduced variability in stimulation of EndoC-*β*H5 spheroids can help overcome limitations of drug screening on human islets. Insulin stimulation and metabolic assays show there is promise for endothelial/beta-cell coculture spheroids as *in vitro* models for T1D research. The two cell types integrate readily, forming robust aggregates that respond to glucose with insulin. Future studies should focus on establishing vascular connectivity with a support scaffold and the immunogenic responses of the heterocellular constructs.

## Data Availability

The datasets used and/or analyzed during the current study available from the corresponding author on reasonable request.

## Nomenclature

### Abbreviations

BMI: Body mass index
BSA: Bovine serum albumin
EDTA: Ethylenediaminetetraacetic acid
GSIS: Glucose-stimulated insulin secretion
HUVEC: Human umbilical vein endothelial cell
IBMIR: Instant blood-mediated inflammation response
IBMX: 3-Isobutyl-1-methylxanthine
IEQ: Islet equivalent
INGAP-P: Islet neogenesis-associated protein-pentadecapeptide
T1D: Type 1 diabetes
TBS: Tris-buffered saline.

## Supplementary Materials

Supplementary Materials Table S1: cell culture groups used for spheroid formation and testing. Table S2: RT-qPCR target genes and mRNA primer sequences. Figure S1: cross-sectional areas of EndoC-*β*H5 and EndoC-*β*H5/HUVEC coculture spheroids, quantified by fluorescent confocal imaging. Figure S2: (A) percent viability of MIN6, EndoC-*β*H5, and EndoC-*β*H5/HUVEC spheroids determined by live/dead staining. (B) Percent reduction of the AlamarBlue metabolic test reagent incubated with MIN6 or MIN6/HUVEC spheroids on days 3, 5, and 8 after formation. Figure S3: cell-specific staining for insulin and CD31 in 1 : 1 (EndoC-*β*H5) spheroid including nuclear staining with DAPI.
