# Supplementary material for: Renewable Human Cell Model for Type 1 Diabetes Research: EndoC-βH5/HUVEC Coculture Spheroids
Source: J Diabetes Res. 2023 Dec 21;2023:6610007. doi: 10.1155/2023/6610007 (PMC10757655; doi:10.1155/2023/6610007)
Supplement: Supplementary Materials — Table S1: cell culture groups used for spheroid formation and testing. Table S2: RT-qPCR target genes and mRNA primer sequences. Figure S1: cross-sectional areas of EndoC-βH5 and EndoC-βH5/HUVEC coculture spheroids, quantified by fluorescent confocal imaging. Figure S2: (A) percent viability of MIN6, EndoC-βH5, and EndoC-βH5/HUVEC spheroids determined by live/dead staining. (B) Percent reduction of the AlamarBlue metabolic test reagent incubated with MIN6 or MIN6/HUVEC spheroids on days 3, 5, and 8 after formation. Figure S3: cell-specific staining for insulin and CD31 in 1 : 1 (EndoC-βH5) spheroid including nuclear staining with DAPI. [file 6610007.f1.docx]

Supporting Information:

Renewable Human Cell Model for Type 1 Diabetes Research:

EndoC-$\beta$H5/HUVEC Coculture Spheroids

James M. Porter^a^, Michael Yitayew^a^ and Maryam Tabrizian^a,b,*^

^a^Dept. of Biological and Biomedical Engineering, Faculty of Medicine and Health Sciences, McGill University, Montreal, QC H3A 0G4, Canada

^b^Faculty of Dental Medicine and Oral Health Sciences, McGill University, Montreal, QC H3A 1G1, Canada
 *Corresponding Author

Table S1. Spheroid Groups

| Cell Types | Cell A (%) | Cell B (%) |
| --- | --- | --- |
| MIN6 | 100 | 0 |
| MIN6/HUVEC | 50 | 50 |
| EndoC-$\beta$H5  EndoC-$\beta$H5/HUVEC  EndoC-$\beta$H5/HUVEC | 100  50  25 | 0  50  75 |

Table S2. RT-qPCR $\beta$-cell gene targets

| Gene | Function | Forward Sequence | Reverse Sequence |
| --- | --- | --- | --- |
| GAPDH | Catalyzes glycolysis, can activate transcription | CAC-CCA-CTC-CTC-CAC-CTT-TG | CCA-CCA-CCC-TGT-TGC-TGT-AG |
| Insulin | Encodes for insulin hormone production | GAA-CGA-GGC-TTC-TTC-TAC-AC | ACA-ATG-CCA-CGC-TTC-TG |
| Glut2  PDX1  MafA | Transmembrane glucose transporter  Transcription Activator  Regulator of insulin gene | CTC-TCC-TTG-CTC-CTC-CTC-CT  ATG-GAT-GAA-GTC-TAC-CAA-AGC  ATTCTGGAGAGCGAGAAGTGCCAA | TTG-GGA-GTC-CTG-TCA-ATT-CC  CGT-GAG-ATG-TAC-TTG-TTG-AAT-AG  CGCCAGCTTCTCGTATTTCTCCTT |


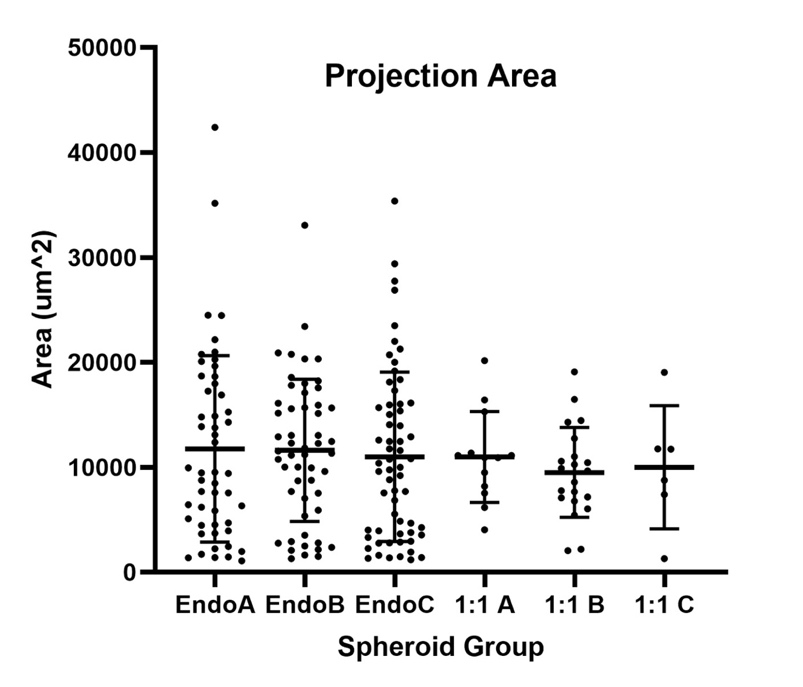


Figure S1:ImageJ quantification of spheroid area, for individual aggregates in each of 3 batches for EndoC-$\beta$H5 and 1:1 EndoC-$\beta$H5/HUVEC coculture spheroids


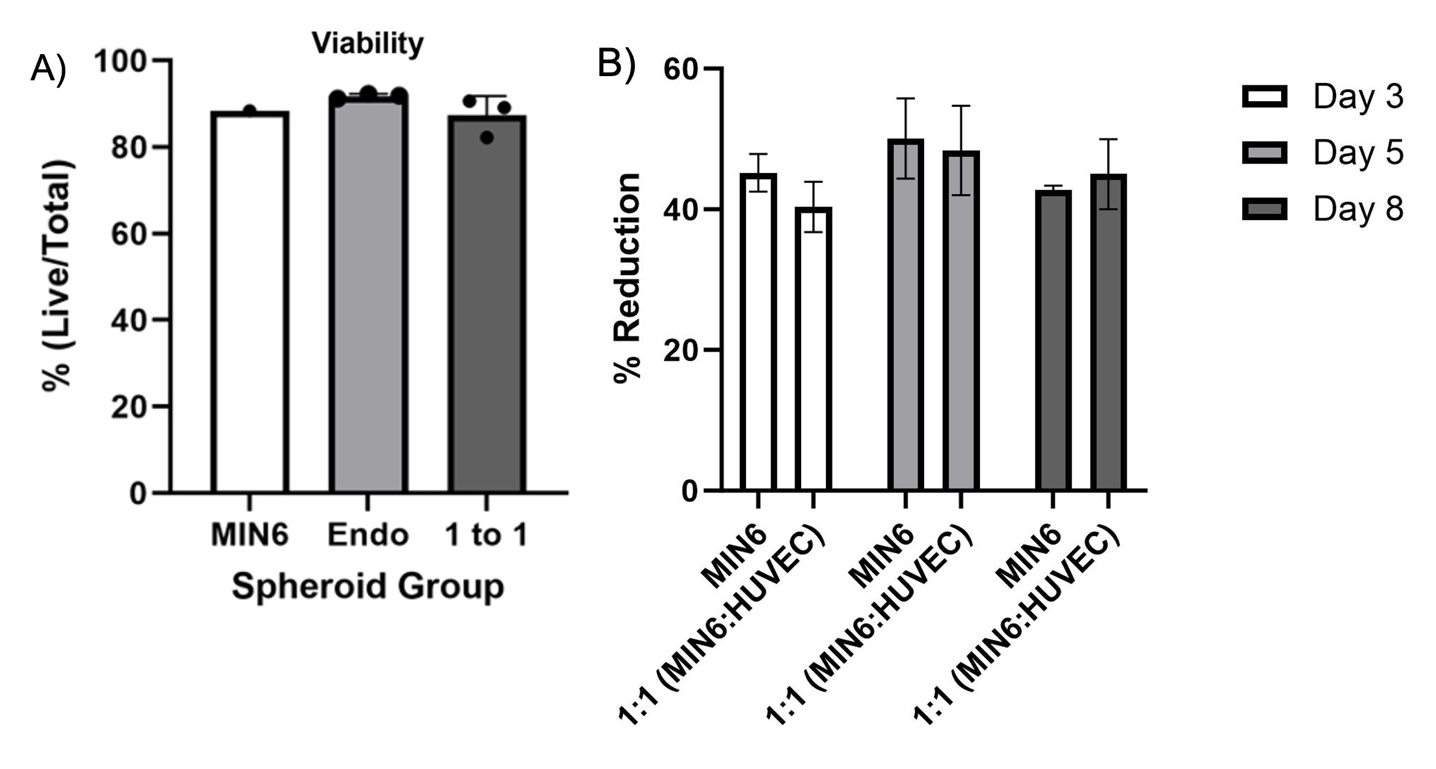


Figure S2: (A) Mean viability shows statistically similar values for MIN6 and EndoC-$\beta$H5 monoculture with 1:1 $\beta$H5/HUVEC coculture spheroids, by one way ANOVA. (B) Metabolic activity of MIN6 and 1:1 MIN6:HUVEC spheroids at days 3, 5 and 8 after formation, as measured by AlamarBlue assay.

Figure S3: Cell-specific staining of 1:1 (EndoC-$\beta$H5:HUVEC) spheroid including nuclear staining with DAPI


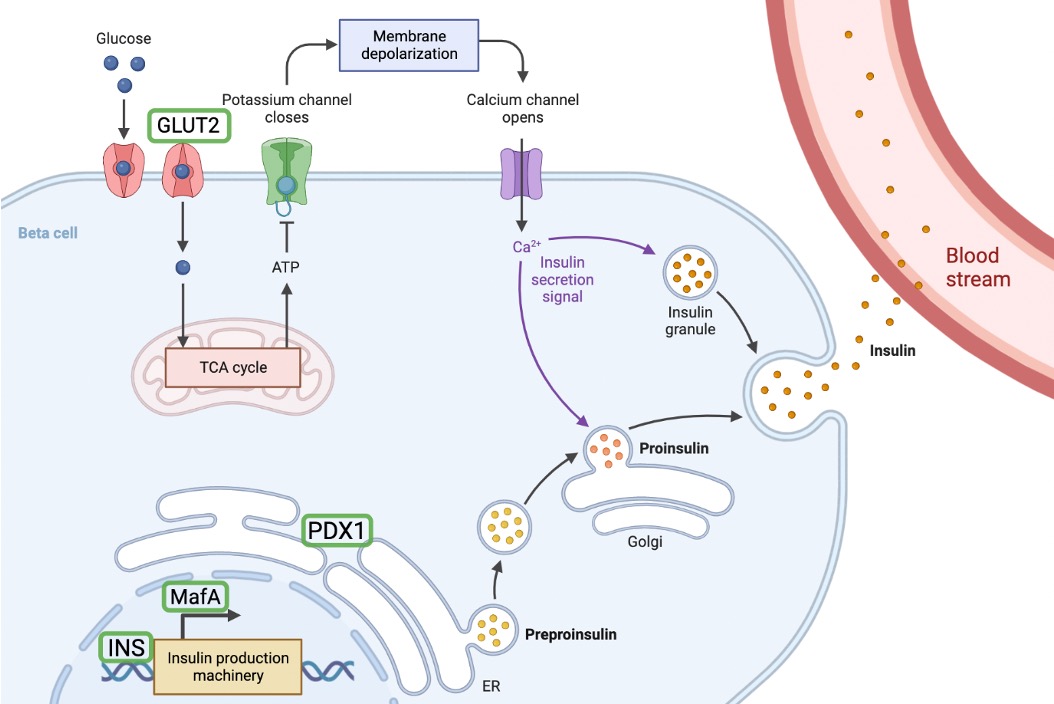


Figure S4: Cell-specific gene targets for RT-qPCR analysis
